# Effect modification of social-context changes on mental disability in China from 1987 to 2006: a multi-level study of 1.9 million people

**DOI:** 10.1186/s12889-017-4066-5

**Published:** 2017-02-13

**Authors:** Zhenjie Wang, Lihua Pang, Ning Li, Chao Guo, Gong Chen, Xiaoying Zheng

**Affiliations:** 1 Institute of Population Research/WHO Collaborating Center on Reproductive Health and Population Science, Peking University, No.5 Yiheyuan Road, Haidian District, Beijing, 100871 People’s Republic of China; 2 Laboratory of Neuroscience and Mental Health, Peking University, No.5 Yiheyuan Road, Haidian District, Beijing, 100871 People’s Republic of China

**Keywords:** Multi-level, Mental disability, China

## Abstract

**Background:**

Very little literature has explored how mental disability in China is connected with inequalities in social and environmental contexts. In the study described herein, we determine whether social-context inequalities were associated with mental disability in China from 1987 to 2006.

**Methods:**

Data were derived from national representative population-based data from the 1987 and 2006 China National Sample Survey on Disability. Both surveys used multistage, stratified random cluster sampling, with a probability proportionate to size, to derive nationally representative samples. A multilevel logistic regression model was applied to estimate the effects of province-specific contextual characteristics on men and women. We also examined the association with mental disability risk stratified by selected covariates. Study populations were (*N* = 698,810) in 1987 and (*N* = 1,260,947) in 2006.

**Result:**

Most of the province-level variables in the 1987 and 2006 surveys were unrelated to mental disability risks in either men or women after controlling for individual characteristics. The age-adjusted prevalence of mental disability nearly doubled among men and women from 1987 to 2006. The effects of the province-specific prevalence of agricultural, forestry, animal husbandry and fishery activities and the percentage of the population age 65 and over significantly lowered the risk of mental disability among women in 1987, by 48 and 32%, respectively. Moreover, the number of health professionals modified the association with per capita gross domestic product (GDP) among women but only in 1987.

**Conclusion:**

To face the challenges of mental disability and interprovincial inequality, the Chinese government should adjust its strategies not only for health-care systems but also to correct for inequalities in interprovincial development; this action may help prevent mental disability.

## Background

Mental health is an essential state of wellbeing [1]. In China, the prevalence of mental disorders has increased rapidly, by approximately 17%, based on a study conducted in four provinces. Moreover, approximately 5.8 million Chinese live with mental disability, and the national prevalence of mental disability increased significantly from 1987 to 2006 during a time of rapid change in the social environment [2–4]. Mental disorders can often be shaped by social inequalities, the economy, and physical environments [5], and these disorders might lead to mental disability. People with mental disorders and disabilities may experience long-term impairments in personal and social functioning.

Mental health, including mental disorders, can be influenced by determinants of the social environment in multiple sectors and at multiple levels [5]. However, previous studies on disability have mainly explored socioeconomic disparities/inequalities on the individual level [5–7]. People with disabilities experience worse socioeconomic outcomes than people without disabilities: they experience higher rates of poverty, have lower employment rates and have less education [6]. They also have unequal access to health-care services and therefore have unmet health-care needs, compared to the general population [6, 7]. However, an individual’s mental health is also associated with local and national factors, such as neighborhood trust and safety, poverty reduction, access to education, and access to health-care, among other factors. These factors are important for two reasons: they influence the risk of mental disorders, and they present opportunities for intervening to reduce this risk [5].

Until now, very little evidence has supported the association between macro-level contexts, such as socioeconomic and health-care development, and mental disabilities, especially taking into consideration twenty years of social changes in China. In this study, we aim to investigate connections between mental disability and inequalities in social development, using two national representative surveys on disabilities [8, 9].

## Methods

### Data source

Data were derived from representative population-based data from the 1987 and 2006 China National Sample Survey on Disability. Both surveys used multistage, stratified random cluster sampling, with a probability proportionate to size, to derive nationally representative samples. The protocol and questions for this survey were reviewed by leading national and international experts, and the sampling scheme was reviewed by experts from the Division of Statistics of the United Nations [8, 9]. The surveys were approved by the State Council and conducted in all province-level administrative regions of mainland China by the Leading Group of the China National Sample Survey on Disability and the National Bureau of Statistics; all survey respondents provided consent to participate in these surveys and clinical diagnosis. Details of the survey’s design and conduct were described elsewhere [4], and these two surveys were comparable [10, 11]. The sampling ratio was 1.50 per 1,000 people for the 1987 survey and 1.93 per 1,000 people for the 2006 survey [8, 9].

### Interviewing procedures and data quality

Two pilot studies were conducted in different provinces before the surveys. Strict quality-control measures were implemented at every step during the surveys, from drafting the sampling frame to field sampling, from filling out of questionnaires to checking the returned forms, and from entering data to checking data quality [8, 9]. During data collection, trained field interviewers used a structured questionnaire to inquire about mental disability. Those who responded “yes” to any of the corresponding questions (“Are you or your family members forgetful? Or do you have difficulties in concentrating? Or can you not control your moods? Or do you have strange behavior that is out of the ordinary? Or are you addicted to alcohol or drugs?”) were referred to designated physicians for further disability screening and confirmation. A designated psychiatrist performed medical examinations and followed diagnostic manuals to make final diagnoses; assess the severity of the disability, if any; and confirm its primary causes. Respondents with multiple positive answers were examined by multiple specialists (a separate doctor for each disability).

After the field investigations were concluded, the teams made home re-visits to conduct surveys in the quarters chosen for post-survey quality checks and calculate errors in the survey overall. The results of the quality checks showed that the omission rate of the resident population was 1.06 per 1,000 people in 1987 and 1.31 per 1,000 people in 2006; the omission rate of the disabled population was 1.16 per 1,000 people in 1987 and 1.12 per 1,000 people in 2006 [8, 9].

### Identification of mental disability

Mental disability cases were defined and classified by the expert committee of the China National Sample Survey on Disability, in both 1987 and 2006, based on the following definition of mental disability: “Mental disability refers to mental disorders lasting more than one year that manifest in cognitive, affective and behavioral disorders that limit one’s daily life and restrict a person’s participation” [8, 9]. The 1987 survey was based on the International Classification of Impairment, Disability and Handicap [12], and the 2006 survey followed the International Classification of Functioning, Disability and Health [13] in the design. All the classifications, screening methods, diagnosing methods, and relevant scales on disabilities were pre-tested in pilot studies with good reliability and validity [8, 9].

### Study variable definition

We defined the status of mental disability as binary, i.e., yes or no. Individual-level variables included age groups as 0–24, 25–49, 50–74 or 75+; gender as male or female; residential area as urban or rural, according to Hu Kou records; ethnicity as Han or other; education level as never attended school, primary school, junior high school or above; marital status as never married, divorced/widowed or married; household size as 1–3, 4–6 or 7–9 (persons per household); living arrangement as living with others or living alone; and current employment status as employed or unemployed. Province-level variables included per capita gross domestic product (GDP); the number of health professionals (per 1,000 people); the proportion of agricultural, forestry, animal husbandry and fishery activities; the percentage of illiterate residents; and the percentage of the population age 65 and older divided into tertile categories as high (≥66.7%), medium (≥33.3% and <66.7%) and low (<33.3%).

### Statistical analysis

A random effects (“multilevel”) logistic regression model was used to estimate the effects of province-level contextual characteristics on mental disability in men and women, respectively. We started by assessing province-to-province differences in mental disability. This task was accomplished by fitting fully unconditional random effects models with random intercepts at the area level. These models allowed us to estimate an interclass cluster coefficient (ICC) that can be interpreted as the proportion of total variance in mental disability that could be attributed to provincial factors. ICCs were statistically significant for men’ and women’ empty models in both the 1987 and 2006 study samples. The following step aimed to control for plausible known individual-level confounders and added area-level variables separately (Mode l-6). Furthermore, stratified analysis was performed with respect to tertiles of GDP and health professionals (high, medium and low). Statistical significance was set at a two-tailed P value of <0.05. Allowing for changes in the age structure of the Chinese population, we calculated the age-adjusted prevalence of disability through direct standardization by using the 1990 Chinese census as the standard [14]. The statistical analyses were performed using SAS v. 9.2 (SAS Institute, Inc., Cary, NC, USA), using the NLMIXED procedure for the multilevel analyses.

## Results

In the twenty-year period considered in this study, the average annual growth of mental disability cases was 8%. The age-adjusted prevalence of mental disability nearly doubled in twenty years. In both survey years, male subjects, rural residents, people living with others and people of Han ethnicity accounted for the majority of cases (Table 1). In addition, the structures of household size and education changed markedly in those twenty years.

**Table 1:** Characteristics of study population in 1987 and 2006

	Total		Male		Female	
	Unweight	Weighted	Unweight	Weighted	Unweight	Weighted
Individual-level variables						
1987						
Sample size	1,398,055	94,6282,249	702,759	475,941,433	695,296	47,0340,816
Age, mean	31.4	31.4 (31.3-31.5)	31.1	31.1 (31.0-31.2)	31.7	31.7 (31.6-31.8)
Residence, %						
Urban	29.4	28.8 (27.2-30.3)	29.1	28.4 (26.9-29.9)	29.8	29.1 (27.6-30.7)
Rural	70.6	71.2 (69.7-72.8)	70.9	71.6 (70.1-73.1)	70.2	70.8 (69.3-72.4)
Ethnicity, %						
Han	91.0	91.5 (90.8-92.3)	91.0	91.6 (90.8-92.3)	90.9	91.5 (90.8-92.3)
Others	9.1	8.5 (7.7-9.2)	9.0	8.4 (7.7-9.2)	9.1	8.5 (7.7-9.2)
Education, %						
Junior high school and above	31.6	31.0 (30.5-31.6)	38.0	37.6 (37.1-38.1)	25.0	24.4 (23.8-24.9)
Primary school	39.0	39.4 (39.0-39.7)	42.7	43.2 (42.8-43.5)	35.2	35.5 (35.2-35.9)
Never attended school	29.4	29.6 (29.1-30.0)	19.3	19.2 (18.8-19.6)	39.8	40.1 (39.5-40.7)
Marital status, %						
Married	53.3	53.2 (53.0-53.4)	51.7	51.5 (51.3-51.7)	55.0	54.8 (54.7-55.0)
Divorced or widowed	5.8	5.8 (5.7-5.9)	4.0	4.0 (3.9-4.1)	7.6	7.6 (7.5-7.7)
Never married	40.9	41.0 (40.8-41.2)	44.3	44.5 (44.3-44.7)	37.4	37.5 (37.3-37.7)
Household size						
1–3	19.8	19.7 (19.4-20.1)	20.4	20.4 (20.0-20.7)	19.2	19.1 (18.7-19.5)
4–6	61.0	61.3 (61.0-61.6)	61.3	61.6 (61.2-61.9)	60.7	61.0 (60.7-61.3)
7–9	19.2	19.0 (18.6-19.4)	18.3	18.1 (17.6-18.5)	20.1	19.9 (19.5-20.4)
Living arrangement						
Living with others	98.7	98.6 (78.6-79.2)	98.4	98.4 (98.3-98.4)	98.9	98.9 (98.9-99.0)
Living alone	1.3	1.4 (1.3-1.4)	1.6	1.6 (1.5-1.7)	1.1	1.1 (1.0-1.1)
Currently employed, %						
Yes	78.8	78.9 (78.6-79.2)	86.7	86.8 (86.6-86.9)	70.8	70.9 (70.4-71.5)
No	21.2	21.1 (20.8-21.4)	13.3	13.2 (13.1-13.4)	29.2	29.0 (28.5-29.6)
2006						
Sample	2,526,134	1,309,468,507	1,280,006	664,280,796	1,246,128	645,187,711
Age, mean	35.8	35.7 (35.6-35.8)	35.2	35.1 (35.0-35.2)	36.5	36.4 (36.3-36.5)
Residence, %						
Urban	33.9	30.8 (29.6-31.9)	32.8	30.1 (28.9-31.2)	34.3	31.4 (30.3-32.6)
Rural	66.1	69.2 (68.1-70.4)	67.2	69.9 (68.8-71.1)	65.7	68.6 (67.4-69.7)
Ethnicity, %						
Han	88.2	90.1 (89.5-90.7)	88.2	90.1 (89.4-90.7)	88.2	90.1 (89.5-90.7)
Others	11.8	9.9 (9.3-10.5)	11.8	9.9 (9.3-10.6)	11.8	9.9 (9.3-10.5)
Education, %						
Junior high school and above	49.8	48.8 (48.3-49.2)	54.9	54.1 (53.7-54.5)	44.5	43.3 (42.8-43.7)
Primary school	31.1	31.7 (31.5-32.0)	31.3	31.9 (31.5-32.2)	30.9	31.6 (31.3-31.9)
Never attended school	19.2	19.5 (19.3-19.7)	13.8	14.0 (12.8-14.2)	24.6	25.1 (24.8-25.5)
Marital status, %						
Married	60.3	60.2 (60.3-60.4)	58.8	58.6 (58.4-58.8)	61.9	61.9 (61.7-62.1)
Divorced or widowed	6.3	6.2 (6.2-6.3)	4.1	4.1 (4.1-4.2)	8.5	8.4 (8.3-8.5)
Never married	33.4	33.6 (33.4-33.7)	37.1	37.3 (37.1-37.5)	29.7	29.7 (29.5-29.9)
Household size						
1–3	44.5	43.4 (42.9-43.9)	45.4	44.4 (43.9-44.9)	43.5	42.4 (41.9-42.9)
4–6	50.7	51.8 (51.4-52.3)	49.9	51.0 (50.6-51.5)	51.4	52.7 (52.2-53.1)
7–9	4.9	4.7 (4.6-4.9)	4.7	4.6 (4.4-4.7)	5.1	4.9 (4.8-5.1)
Living arrangement						
Living with others	97.5	97.5 (97.4-97.6)	97.4	97.4 (97.4-97.5)	97.6	97.7 (97.6-97.7)
Living alone	2.5	2.5 (2.4-2.5)	2.6	2.7 (2.5-2.6)	2.4	2.3 (2.2-2.4)
Currently employed, %						
Yes	55.3	56.1 (55.9-56.4)	59.8	60.2 (60.0-60.4)	50.8	51.9 (51.6-52.3)
No	44.7	43.9 (43.6-44.1)	40.2	39.8 (39.6-40.0)	49.2	48.1 (47.7-48.4)
Province-level variables	1987				2006	
	Median	Interquartile range			Median	Interquartile range
Per capita gross domestic product (yuan)	756	641-1098			13313	10798-21788
Number of health professional in per 1,000 people	3.4	2.8-4.7			3.7	3.0-4.5
Proportion of agriculture, forestry, animal husbandry and fishery (%)	77	62.5-80.2			1.9	1.1-5.3
Illiterate (%)	26.2	20.5-30.7			9.2	5.2-11.3
Percentage of aged 65 and older (%)	5.5	5.0-6.0			9	8.1-10.6

**Fig. 1:**
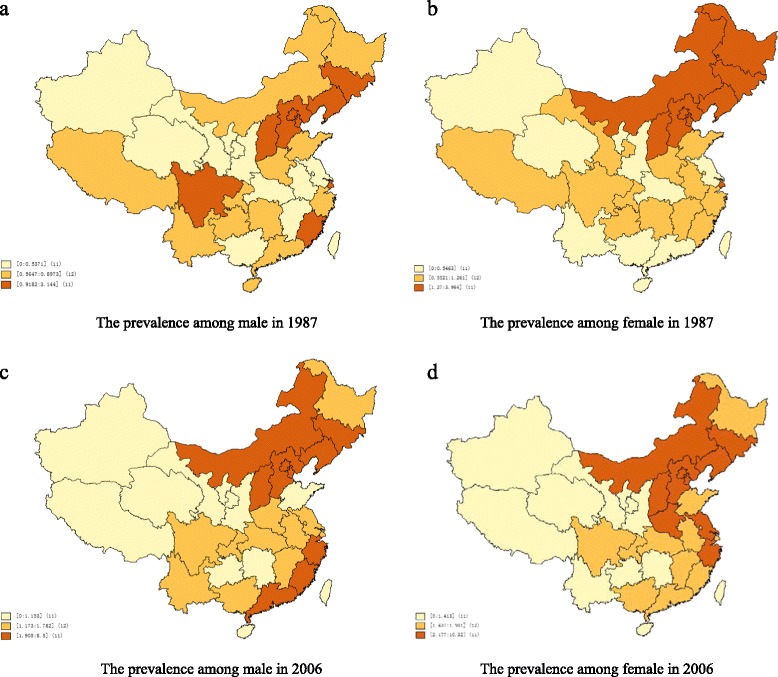
Distribution of age-adjusted mental disability prevalence in China

**Fig. 2:**
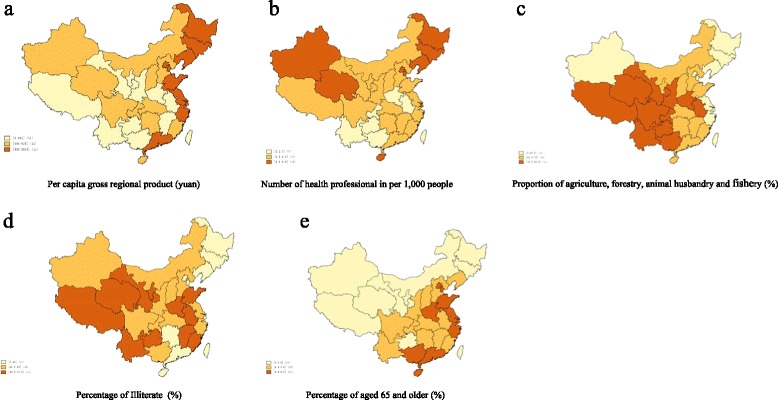
Tertiles of province-level characteristics distribution in China in 1987

**Fig. 3:**
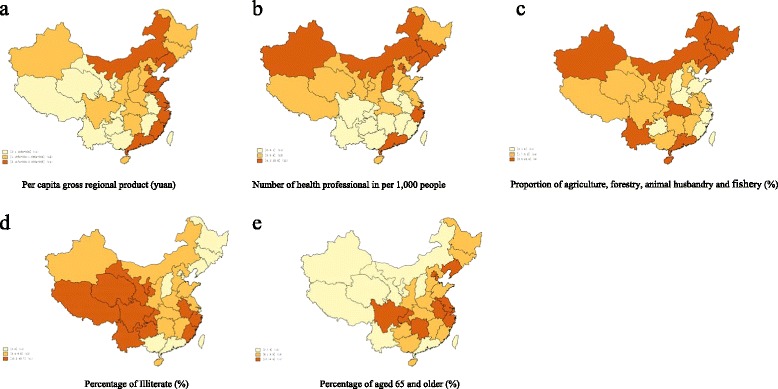
Tertiles of province-level characteristics distribution in China in 2006

Spatial distributions of mental disability prevalence and province-level characteristics are presented in Figs. 1, 2 and 3. The average annual growth of GDP was 16% from 1987 to 2006. However, the average annual growth in the number of health professionals (per 1,000 people), which counted health professionals in all types of health-care facilities, was only 0.4% in the same period. Furthermore, the spatial distribution of mental disability prevalence among women was correlated with GDP in 1987 and with the number of health professionals (per 1,000 people) in both 1987 and 2006. However, we did not observe similar associations among men in either 1987 or 2006.

Associations between provincial variables and mental disability by gender in 1987 and 2006 are presented in Tables 2 and 3. Compared to the highest categories, including the proportion of agricultural, forestry, animal husbandry and fishery activities and the percentage of residents age 65 and over, the risk of mental disability in women connected with the lowest categories significantly decreased, by 48 and 32%, respectively, in 1987. However, we did not observe the same associations among women in 2006.

**Table 2:** Multilevel logistic regression models for province-level variables and mental disability in 1987^a^

	1987, OR (95% CI)					
	Model 1	Model 2	Model 3	Model4	Model 5	Model6
Male						
Per capita gross domestic product						
High	Reference					Reference
Medium	1.05 (0.73-1.37)					0.86 (0.52-1.19)
Low	1.00 (0.70-1.31)					0.64 (0.29-1.00)
Number of health professional in per 1,000 people						
High		Reference				Reference
Medium		0.83 (0.57-1.10)				0.74 (0.49-0.98)
Low		1.19 (0.90-1.48)				1.14 (0.80-1.48)
Proportion of agriculture, forestry, animal husbandry and fishery (%)						
High			Reference			Reference
Medium			1.03 (0.77-1.35)			0.92 (0.62-1.22)
Low			0.90 (0.64-1.16)			0.68 (0.27-1.09)
Illiterate (%)						
High				Reference		Reference
Medium				1.06 (0.72-1.39)		0.84 (0.53-1.16)
Low				0.93 (0.66-1.20)		0.85 (0.47-1.23)
Percentage of aged 65 and over (%)						
High					Reference	Reference
Medium					1.01 (0.67-1.35)	0.81 (0.48-1.13)
Low					0.87 (0.62-1.13)	0.85 (0.55-1.15)
ICC	0.024	0.034	0.021	0.04	0.024	0.014
Female						
Per capita gross domestic product						
High	Reference					Reference
Medium	1.04 (0.73-1.35)					0.98 (0.63-1.33)
Low	1.27 (0.89-1.65)					0.96 (0.48-1.46)
Number of health professional in per 1,000 people						
High		Reference				Reference
Medium		0.75 (0.50-1.00)				0.65 (0.45-0.85)
Low		0.96 (0.71-1.22)				0.77 (0.55-0.98)
Proportion of agriculture, forestry, animal husbandry and fishery (%)						
High			Reference			Reference
Medium			0.67 (0.48-0.86)			0.69 (0.48-0.90)
Low			0.86 (0.60-1.16)			0.52 (0.23-0.80)
Percentage of illiterate (%)						
High				Reference		Reference
Medium				0.86 (0.58-1.14)		0.79 (0.52-1.07)
Low				0.86 (0.60-1.16)		0.95 (0.55-1.35)
Percentage of aged 65 and over (%)						
High					Reference	Reference
Medium					1.08 (0.70-1.46)	0.72 (0.45-0.99)
Low					1.01 (0.70-1.32)	0.68 (0.47-0.90)
ICC	0.025	0.025	0.019	0.027	0.028	0.011

*Abbreviations*: *CI* confidence interval, *OR* odds ratio

^a^ Model 1: adjustment for per capita gross domestic product and individual-level variables; Model 2: adjustment number of health professional per 1,000 people and individual-level variables; Model 3: adjustment proportion of agriculture, forestry, animal husbandry and fishery and individual-level variables; Model 4: adjustment percentage of illiterate and individual-level variables; Model 5: adjustment percentage of aged 65 and over and individual-level variables; Model 6: adjustment for area-level variables and individual-level variables

**Table 3:** Multilevel logistic regression models for province-level variables and mental disability in 2006^a^

	2006, OR (95% CI)					
	Model 1	Model 2	Model 3	Model4	Model 5	Model6
Male						
Per capita gross domestic product						
High	Reference					Reference
Medium	0.94 (0.71-1.18)					0.93 (0.67-1.18)
Low	1.03 (0.78-1.28)					0.93 (0.56-1.30)
Number of health professional in per 1,000 people						
High		Reference				Reference
Medium		0.92 (0.70-1.14)				0.88 (0.59-1.16)
Low		1.15 (0.89-1.42)				1.13 (0.71-1.56)
Proportion of agriculture, forestry, animal husbandry and fishery (%)						
High			Reference			Reference
Medium			1.08 (0.82-1.34)			1.06 (0.77-1.35)
Low			1.06 (0.80-1.32)			1.07 (0.77-1.36)
Illiterate (%)						
High				Reference		Reference
Medium				0.93 (0.70-1.16)		0.98 (0.66-1.30)
Low				0.87 (0.66-1.08)		0.91 (0.64-1.19)
Percentage of aged 65 and over (%)						
High					Reference	Reference
Medium					1.10 (0.82-1.38)	1.21 (0.89-1.53)
Low					1.05 (0.79-1.31)	1.12 (0.82-1.42)
ICC	0.020	0.018	0.020	0.019	0.020	0.016
Female						
Per capita gross domestic product						
High	Reference					Reference
Medium	1.07 (0.80-1.33)					1.11 (0.79-1.42)
Low	0.96 (0.73-1.20)					1.12 (0.67-1.57)
Number of health professional in per 1,000 people						
High		Reference				Reference
Medium		0.87 (0.66-1.09)				0.84 (0.56-1.11)
Low		1.00 (0.76-1.24)				0.98 (0.60-1.35)
Proportion of agriculture, forestry, animal husbandry and fishery (%)						
High			Reference			Reference
Medium			1.00 (0.76-1.24)			1.07 (0.78-1.36)
Low			1.07 (0.81-1.33)			1.03 (0.75-1.31)
Illiterate (%)						
High				Reference		Reference
Medium				1.22 (0.93-1.51)		1.31 (0.87-1.74)
Low				1.19 (0.91-1.47)		1.20 (0.83-1.56)
Percentage of aged 65 and over (%)						
High					Reference	Reference
Medium					0.89 (0.67-1.12)	0.93 (0.68-1.18)
Low					0.87 (0.66-1.06)	0.92 (0.67-1.17)
ICC	0.021	0.020	0.021	0.019	0.020	0.017

*Abbreviations*: *CI* confidence interval, *OR* odds ratio

^a^ Model 1: adjustment for per capita gross domestic product and individual-level variables; Model 2: adjustment number of health professional per 1,000 people and individual-level variables; Model 3: adjustment proportion of agriculture, forestry, animal husbandry and fishery and individual-level variables; Model 4: adjustment percentage of illiterate and individual-level variables; Model 5: adjustment percentage of aged 65 and over and individual-level variables; Model 6: adjustment for area-level variables and individual-level variables

We further analyzed the association of GDP with mental disability, stratified by the number of health professionals (per 1,000 people) (Table 4). We found that, among women in 1987, GDP status tended to be associated with mental disability risk when the number of health professionals (per 1,000 people) was lowest. In other words, in 1987, the risk seemed to increase for female subjects who lived in provinces with the lowest status of variables. However, this association changed to a bell-shaped trend among women in 2006.

**Table 4:** Multilevel logistic regression models for province-level variables interaction effects on mental disability^a^

	Per capita gross domestic product, OR (95% CI)			
	High	Medium	Low	*P*_*trend*_
1987				
Male				
Number of health professional in per 1,000 people				
High	Reference	0.84 (0.57-1.11)	0.96 (0.53-1.39)	0.86
Medium	0.69 (0.40-0.99)	1.31 (0.59-2.03)	0.63 (0.34-0.93)	0.98
Low	1.20 (0.53-1.86)	1.20 (0.77-1.63)	1.03 (0.72-1.35)	0.37
Female				
Number of health professional in per 1,000 people				
High	Reference	0.89 (0.59-1.20)	1.33 (0.70-1.95)	0.42
Medium	0.64 (0.35-0.94)	0.87 (0.35-1.39)	0.82 (0.43-1.21)	0.43
Low	0.62 (0.24-0.99)	0.88 (0.54-1.23)	1.09 (0.73-1.46)	0.01
2006				
Male				
Number of health professional in per 1,000 people				
High	Reference	0.92 (0.63-1.20)	NA	NA
Medium	1.01 (0.71-1.49)	1.03 (0.76-1.30)	0.72 (0.47-0.97)	0.16
Low	1.11 (0.58-1.64)	1.15 (0.74-1.55)	1.21 (0.93-1.49)	0.57
Female				
Number of health professional in per 1,000 people				
High	Reference	1.03 (0.71-1.35)	NA	NA
Medium	1.09 (0.71-1.47)	1.02 (0.76-1.29)	0.77 (0.51-1.04)	0.20
Low	0.60 (0.31-0.89)	1.46 (0.95-1.97)	1.08 (0.83-1.33)	0.33

*Abbreviations: CI* confidence interval, *OR* odds ratio, *NA* not available

^a^ adjustment for individual-level variable

## Discussion

For twenty years, China underwent rapid development and change, with an economic growth rate of 7.5-13.0% per year [15]. However, the growth in wealth has not been equitably distributed, resulting in an increasing gap between the rich and the poor. It is evident that those with the greatest socioeconomic disadvantages are often those with the highest mental health-care needs [16]. The prevalence of mental disability as diagnosed based on performance nearly doubled among both men and women during that time period. One potential reason for the upward trend in prevalence might be changes in attitudes about mental health because of increasing public awareness and changes in diagnostic criteria [17]. Although the awareness of mental health is improving, the increase in prevalence of mental disability might also be attributable to the current underdeveloped status of the mental-health service system in China. Firstly, 44.8% of the urban population and 79.1% of the rural population did not have any health-care insurance in 2006 [18]. In mainland China, financial expenditures on the health-care system, as a percentage of the gross domestic product (approximately 5% in recent years), were much smaller than in HK, where the annual government expenditure on health-care increased 40% from 2007 to 2012 [19]. Secondly, there is no national Mental Health Act in China, and China has not yet given the mental-health service system enough priority [20, 21]; there are only 1.3 psychiatrists and 2.1 psychiatric nurses per 100,000 people in China [22]. The slow development of specialized training and treatment for mental disorders and culturally rooted stigmas about mental disorders are also barriers to the improvement of the mental health status of the Chinese population [20, 21].

Very few studies have explored the associations between macro-level context characteristics and mental disability, but similar research has explored the association between macro-level contexts, such as metropolitan area and county, and individual health outcomes [23–25]. These studies suggested that state-level income inequality was associated with self-rated health after controlling for individual demographic factors [24]. In addition, lower levels of geographical aggregation have produced mixed findings for self-rated health in multi-level studies in the US [25]. In other developed countries, the evidence was similarly mixed [23]. For mental disorders, socioeconomic factors and physical environments also greatly shape a population’s mental health at different stages of life. Risk factors for mental disorders were heavily associated with social inequalities, whereby the greater the inequality, the higher the inequality in risk [5]. In China, interprovincial inequality increased in the 1990s but was relatively stable from the late 1990s to 2004 [26]. Existing research has found that both individual and province-level factors contribute to health inequalities in China [27, 28], with province-level effects reflecting regional diversity in welfare provision and the demographic and socioeconomic composition of the population. Another recent Chinese study suggested that the difference in health outcomes between provinces remained substantial even after controlling for a number of individual and household characteristics [27]. In the current study, province-level characteristics, such as the proportion of agricultural, forestry, animal husbandry and fishery activities and the percentage of residents age 65 and over, substantially influenced mental disability among women in 1987 after controlling for individual variables. The proportion of agricultural, forestry, animal husbandry and fishery activities was negatively correlated with GDP (Pearson correlation coefficient: −0.91 *P* <0.01), which suggested that the proportion of agricultural, forestry, animal husbandry and fishery activities could present an angle for economic development, although GDP was not related to the risk of mental disability. The percentage of the population age 65 and over positively correlated with the number of health professionals per 1,000 people (Pearson correlation coefficient: 0.46, *P* <0.01), which suggested that improvements in health-care might prevent the development of mental disability among the aged population. However, we did not observe a similar result in 2006. This may be because social inequities were more serious in 1987 than in 2006, or this result might be due to chance. In this study, however, we did not observe significant associations between most provincial characteristics and the risk of mental disability among men and women in either 1987 or 2006. One possible explanation is that most types of mental disability collected in these surveys were more genetic or biological than environmental. Therefore, these disorders were relatively less environmentally sensitive. However, neuropsychiatric disorders have proved sensitive to macro-environmental change [29]. In one previous study, mental disorders, such as depressive disorders and alcohol dependency, were more prevalent in rural areas than in urban areas in China [30]. Because we analyzed the association between macro-level factors and mental disability risk, not specific types of mental disability, our result might neglect significant associations between macro-level factors and specific types of mental disability. These issuesshould be taken into consideration for further research.

Unlike previous studies that only observed associations between macro-level or individual-level characteristics and health outcomes, we further examined the effects of province-level variables on mental disability among men and women in each survey year. It is a unique finding that the influence of GDP was more evident among women within the lowest categories of the number of health professionals, especially in 1987. There was no existing explanation for the unique result with regard to the effect of the number of health professionals. However, we observed the differences on spatial distributions of province-level variables, which might be one plausible reason for this interesting result. Another possible reason was that province-level contexts might have an impact through different mechanisms on the increased risk of mental disability in Chinese women.

This study has provided a broad understanding of mental disability and its relationship with province-level aspects of development. Moreover, the current study used a large representative survey, which covered all the provincial administrative areas in mainland China. In addition, every subject in the selected households was interviewed face to face. A disability screening was conducted by the interviewers, and those suspected to be disabled were then examined and diagnosed by doctors. The present study has some weaknesses; for example, the 1987 survey classified disability using the international classification of impairments, disabilities, and handicaps [12], and the 2006 survey used the international classification of functioning, disability and health [13]. But both surveys employed the Chinese word “Canji,” which means both handicap and disability, and that helps to maintain the consistency of the definition used in the surveys. The more stringent definition of disability used in both surveys caused a low prevalence of mental disability compared with other research, which should serve to inform future studies. Moreover, the mental disorder classification in both surveys should be viewed with caution, although these classifications were comparable [10, 11]. In addition, standardized quality-control schemes were in place during the field implementation, such as training of the interviewers, and the returned survey responses were crosschecked by contacting survey participants, resulting in little response bias.

## Conclusions

Considering that China is undergoing social and economic transition and experiencing regional inequality, these results will be beneficial for understanding the growth of mental disability and the importance of resource redistribution in China. Furthermore, our results can help the government adjust its strategies to improve individual, community, provincial and national health-care systems and to prevent mental disability and/or improve the lives of people with mental disabilities.
